# DAB2IP in cancer

**DOI:** 10.18632/oncotarget.6501

**Published:** 2015-12-08

**Authors:** Liang Liu, Cong Xu, Jer-Tsong Hsieh, Jianping Gong, Daxing Xie

**Affiliations:** ^1^ Tongji Cancer Research Institute, Tongji Hospital, Tongji Medical College in Huazhong University of Science and Technology, Wuhan, Hubei 430030, China; ^2^ Department of Gastrointestinal Surgery, Tongji Hospital, Tongji Medical College in Huazhong University of Science and Technology, Wuhan, Hubei 430030, China; ^3^ Department of Gastroenterology, Tongji Hospital, Tongji Medical College in Huazhong University of Science and Technology, Wuhan, Hubei 430030, China; ^4^ Department of Urology, University of Texas Southwestern Medical Center, Dallas, TX 75390, USA

**Keywords:** DAB2IP, AIP1, cancer, tumor suppressor

## Abstract

DOC-2/DAB2 is a member of the disable gene family that features tumor-inhibiting activity. The DOC-2/DAB2 interactive protein, DAB2IP, is a new member of the Ras GTPase-activating protein family. It interacts directly with DAB2 and has distinct cellular functions such as modulating different signal cascades associated with cell proliferation, survival, apoptosis and metastasis. Recently, DAB2IP has been found significantly down regulated in multiple types of cancer. The aberrant alteration of DAB2IP in cancer is caused by a variety of mechanisms, including the aberrant promoter methylation, histone deacetylation, and others. Reduced expression of DAB2IP in neoplasm may indicate a poor prognosis of many malignant cancers. Moreover, DAB2IP stands for a promising direction for developing targeted therapies due to its capacity to inhibit tumor cell growth *in vitro* and *in vivo*. Here, we summarize the present understanding of the tumor suppressive role of DAB2IP in cancer progression; the mechanisms underlying the dysregulation of DAB2IP; the gene functional mechanism and the prospects of DAB2IP in the future cancer research.

## 1 INTRODUCTION

DOC2/DAB2 is a tumor suppressor gene associated with ovarian [1, 2], prostate [3, 4] and mammary cancer [5] as well as choriocarcinoma [6]. Using a yeast two-hybrid system, we first identified DAB2IP as a DOC-2/DAB2 interactive protein with several potential functional domains [7]. Its key feature is the Ras GAP homology domain with functional impact on Ras-mediated signal transduction and growth inhibition. In addition, the C2 domain within DAB2IP is reported to bind with Apoptosis signal-regulating kinase 1 (ASK1), and is called ASK1 Interacting Protein (AIP1) [8]. With more and more research available, DAB2IP function is that of a tumor suppressor in tumor cell growth, metastasis, and other aspects in cancer progression. Altered DAB2IP gene expression often detected in cancers is due to epigenetic silencing [9]. In prostate cancer, DAB2IP expression was repressed by promoter methylation and histone modification [10, 11]. Given that DAB2IP appears to have a significantly important role in tumorigenesis and metastasis, we review the current advances about the role of DAB2IP on cancer development, the molecular mechanisms leading to aberrant expression of DAB2IP, and its potential as a therapeutic target.

## 2 The structure of DAB2IP

The DAB2IP is located on human chromosome 9q33.1–q33.3 and spans approximately 96 kb with 15 exons and 14 introns [12]. It is highly polymorphic, 1457 polymorphisms and two different common transcripts have been reported in this gene. Several reports indicate the genetic variation of DAB2IP is associated with the risk of cancer [13–15].

**Figure 1:**
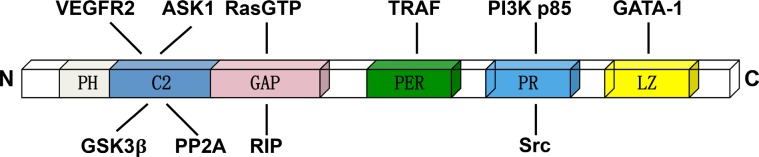
The domain structure of DAB2IP and relative binding proteins The C2 domain can interacts with ASK1, GSK3β and VEFGR2. The GAP domain is the critical binding domain for RasGTP, PP2A and RIP. The PER domain can interacts with TRAF. The PR domain interacts with PI3K p85 subunit and Src, while the LZ domain interacting with the transcription factor GATA-1.

DAB2IP consists of several conserved structural domains: the pleckstrin homology (PH) for membrane targeting, PKC-conserved region 2 (C2) for interactions with ASK1, and Ras-GTPase activating protein (GAP) domain for inhibition of Ras signaling, the C-teminal period-like (PER) domain for inhibition of transcription factor NF-κB, a proline-rich (PR) region for inhibition of PI3K-Akt survival pathway, and aleucine-zipper motif that can inhibit the transcription factor of CD117 [7, 9, 16–20] (Figure 1).

## 3 The regulation of DAB2IP gene expression

### 3.1 Transcriptional regulation of DAB2IP gene

Normal prostatic epithelial cells have elevated hDAB2IP mRNA levels compared with PCa cells, which correlate with increased hDAB2IP promoter activity [7, 12]. This indicates that transcriptional regulation of hDAB2IP may be responsible for the down-regulation of hDAB2IP expression in PCa cells.

The core promoter sequence hypermethylation and histone deacetylation cooperatively silence the DAB2IP gene expression in prostate cancer [10, 12] and other cancer types [21–24]. This results in histone deacetylase inhibitor (TSA-) and hypoemethylation agent (5′-Aza-) acting cooperatively increasing DAB2IP expression in PCa cells [10].

Human enhancer of Zeste homolog gene (Ezh2) encodes a histone lysine methyltransferase [25] and is a predictor of poor outcome for post-prostatectomy in clinically localized PCa [26]. We have demonstrated that the pattern of hDAB2IP gene expression exhibited an inverse correlation with that of Ezh2 in PCa [11]. Moreover, another study shows that gene fusion of DAB2IP may be another mechanism for disrupting DAB2IP function in myeloid leukemia [27].

Recently, Wang J et al. proved that the linear complex of Ezh2/HDAC1/Snail contributed to DAB2IP silencing in colorectal carcinoma (CRC) which could accordingly inducing Snail expression. Thus, the positive feedback loop between snail and DAB2IP forms to promotes invasion and metastasis in CRC [28].

### 3.2 Proteasome-mediated DAB2IP protein degradation

The ubiquitin proteasome system (UPS) is involved in various physiological responses. E3 ligases, the most specific enzymes of unbiquitnation system, participates in the development of cancer [29]. The largest family of E3 ubiquitin ligases consist of four structural and functional components: a substrate-recognizing F-box protein, an adaptor protein SKP1, scaffold protein Cullin (CUL-1, –2, –3, –4A, –4B, –5, and –7) and two RING proteins, RBX1/ROC1 and RBX2/ROC2, also known as SAG (sensitive to apoptosis gene) [30]. Inuzuka et al. found that DAB2IP could interact with Cullin-1 and Cullin- 4A. Cullin-1 and associated F-box protein that directs DAB2IP degradation [31].

S-phase-associated kinase protein-2 (Skp2) is a member of the Skp, Cullin, F-box-containing complex and is an ubiquitin E3 ligase [32]. Our group discovered the Skp-2 mediated UPS played an important role in regulating DAB2IP protein expression post-translation in both immortalized normal prostate epithelial and cancer cells [9]. We further demonstrated that N-terminal end of DAB2IP, particularly the C2 and GAP domains, interacts with Skp2 as a major ubiquitination site. Interestingly, DAB2IP can also regulate Skp2 protein stability in normal or benign cells. Since DAB2IP can deactivate Akt through suppressing PI3K pathway [19] and activating Akt is known to prevent Skp2 degradation [33], DAB2IP is able to modulate Skp2 protein degradation through Akt pathway. The reciprocal regulation between DAB2IP and Skp2 is involved in the growth of prostatic epithelia both *in vitro* and *in vivo*, which represents a unique homeostatic balance.

## 4 Mechanisms of DAB2IP function

**Figure 2:**
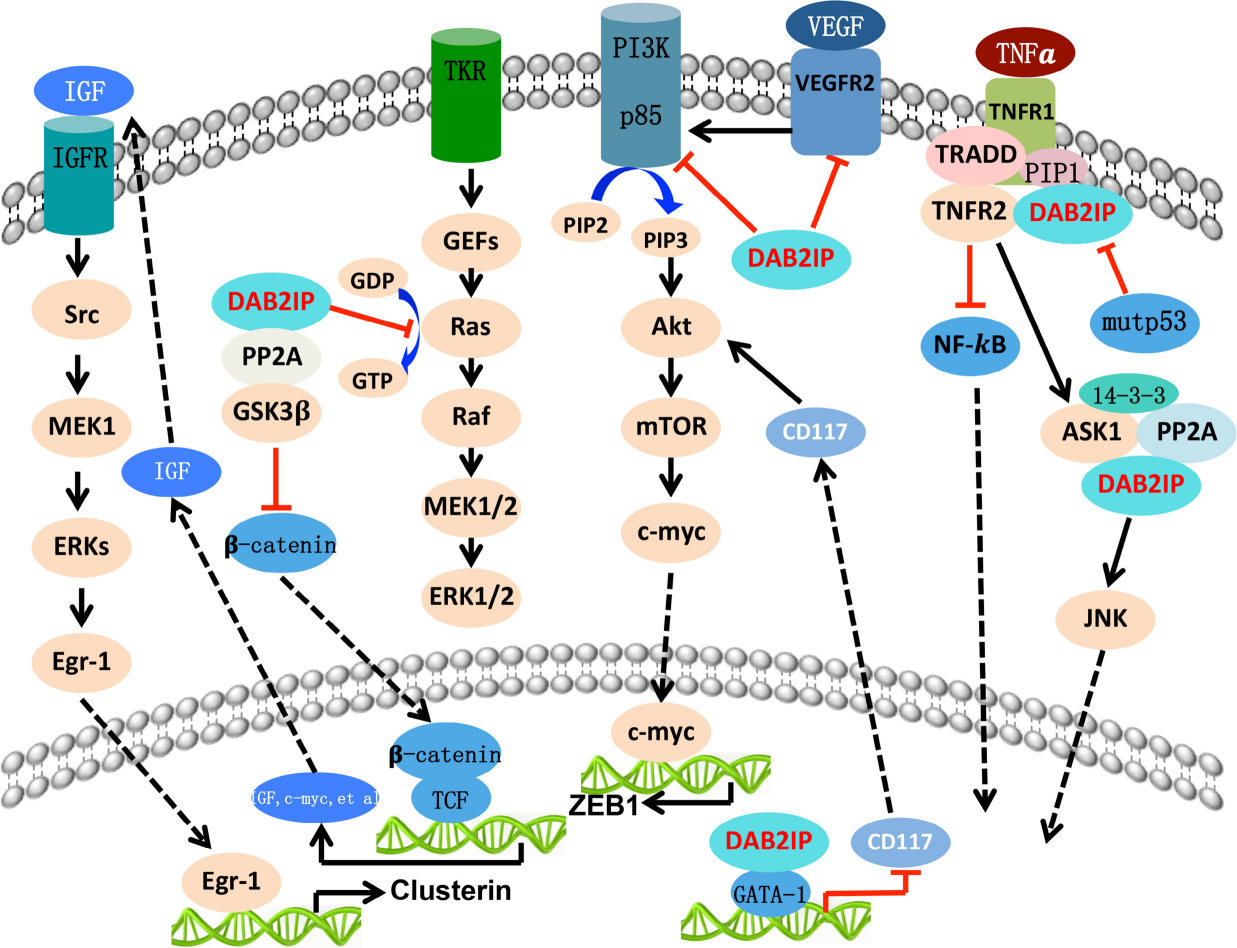
The biologic function of DAB2IP with different signal pathway DAB2IP function as a platform protein and exerts its tumor-supressing acitvity by targeting various critical signal pathways. Overall, DAB2IP inhibit the signaling leading to cell growth, epithelial-to-mesenchymal transition (EMT), angiogenesis, cancer stemness, and autophagy. However, DAB2IP facilitate the process of cell apoptosis, chemoresistance, and radioresistance process.

DAB2IP has been implicated in the regulation of a diverse array of biological processes including proliferation [7], apoptosis [19], epithelial-to-mesenchymal transition (EMT) [34], cancer stem cell (CSC) [35], autophagy [36] and so on, and will be illustrated as follows (Figure 2).

### 4.1 Cell survival and apoptosis

Coordination and balance between cell survival and apoptosis is crucial for normal development and homeostasis of multicellular organisms [37]. If this balance is disrupted, the result could be a variety of diseases including cancer, autoimmune and neurodegenerative conditions [38, 39]. Similar to other signaling pathway regulation, Cell proliferation and apoptosis can be activated by activation of growth-factor receptor and/or binding of integrin to specific extracellular-matrix molecules during cell adhesion.

#### 4.1.1 Ras/Raf/MEK/ERK (MAPK) signaling

In response to extracellular stimuli (growth factors, cytokines, stress, etc.), RAS/RAF/MEK/ERK (MAPK) cascades are activated and mediate both physiological and pathological responses in mammalian cells and tissues [40]. This signaling is activated by replacement of GDP with GTP, whereas GTPase-activating proteins (GAPs) stimulate GTP hydrolysis to GDP [41, 42]. In many human cancers, the Ras pathway is commonly up-regulated or activated through typically to render Ras consititutively GTP-bound, resulting in activation of downstream effector pathway in the absence of extracellular stimuli [43].

Given the fact that DAB2IP has Ras GAP homology domain, it is not surprising that DAB2IP appears to be a tumor suppressor gene since RasGAPs' role includes promoting GTPase inactivation and is often inactivated in cancers [44–49]. Wang et al. showed that DAB2IP could interact with the N-terminal domain of DAB2 protein directly and functions as a Ras GAP *in vivo* and *in vitro* [7].

#### 4.1.2 TNF signaling

Tumor necrosis factor (TNF) can trigger two seemingly opposing major cellular responses: it can induce gene activation and cell death [50]. Once TNF binds to TNFR1, receptor trimerization and formation of the TNF receptor signaling complex happens. This is followed by the signaling molecules TNFR-associated death domain protein (TRADD) and receptor interacting kinase 1 (RIP1) being recruited. TRADD then serves as an assembly platform for binding of TNFR-associated factor 2 (TRAF2), which in turn recruits mitogen-activated protein kinase kinasekinase (MAP3K) and IκB kinase complex (IKK), leading to activation of NF-κB and JNK pathways, respectively [51–53].

Zhang et al. [54] reported that DAB2IP served as a transducer of TRAF2 in participation of TNF signaling. DAB2IP remains an inactive form complex with TNFR1 via its PH/C2 domain. In response to TNF, DAB2IP dissociates from TNFR1 with concomitant of DAB2IP to cytoplasm and forms a complex with TRADD, RIP1, TRAF2, and ASK1. Moreover, this complex is formed after 15 minutes in response to TNF but dissociated by 60 minutes. More importantly, DAB2IP associates with the effector domain (the RING finger) of TRAF2 through its PER domain and mediates TNF/TRAF2-induced ASK1-JNK activation while inhibiting IKK-NF-κB signaling.

#### 4.1.3 ASK1-JNK signaling

As mentioned above, apoptosis signal-regulating kinase 1 (ASK1) is a member of MAP3K family and activates both SEK1-JNK and MKK3/MKK6-p38 signaling cascades [55–58], which plays critical role in signal transduction of apoptosis [59–61].

In searching ASK1 regulatory proteins, DAB2IP was identified as a candidate. The lysine-rich cluster within the C2 domain within DAB2IP is critical for ASK1 binding [8]. Different with the association of ASK1 and 14–3–3, DAB2IP binds preferentially to dephosphorylated ASK1 at Ser967, leading to ASK1 activation, ASK1-induced JNK activation, and EC apoptosis. Moreover, GAP activity toward Ras could inhibit EGF-induced ERK activation [7], so the association of DAB2IP with ASK1 and the GAP activity of DAB2IP are both required for AIP1-enhanced ASK1 activity [8].

Though DAB2IP itself is not a phosphatase protein, DAB2IP could bind phosphatase PP2A through the C2 domain [18]. Also, the functional significance of PP2A-DAB2IP complex is demonstrated by a synergistic effect of PP2A and DAB2IP on activation of ASK-JNK signaling and EC apoptosis. As a result, DAB2IP functions as a scaffold protein in TNF-induced recruitment of PP2A to ASK1 complex, leading to dephosphorylation of ASK1 at pSer967 and activation of ASK1-JNK signaling.

Moreover, RIP in the DAB2IP complex mediates TNF induced DAB2IP phosphorylation at 14–3–3-binding site (Ser604). Moreover, RIP1 associates with the GAP domain of DAB2IP and synergizes DAB2IP-mediated JNK/p38 MAPK activation leading to EC apoptosis [17].

Licio Collavin and his research team further demonstrated that DAB2IP can be functionally inactivated by physical interaction with mutant p53 proteins in the cytoplasm. This mutp53/DAB2IP interaction interferes TNF-induced signaling complexes that activate the ASK1/JNK axis, thereby promoting activation of NF-κB [62].

#### 4.1.4 PI3K/Akt signaling

The phosphatidylinositol 3-kinase/protein kinase-B/mammalian target of rapamycin (PI3K/AKT/mTOR) signaling cascade is one of the most important intracellular pathways and is frequently activated in diverse cancers [63, 64]. In mammals, class I PI3K is divided into class I A group with its 110-kDa catalytic subunit (p110) and an 85-kDa regulatory subunit (p85) [65]. The p85 stabilizes p110 in the steady state [66]. The current view is that pre-formed, inactive p85-p110 complex is present in the cytoplasm of resting cells and poised for activation in response to appropriate cues [67]. The AKT, as the downstream of PI3K, is able to regulate many biological processes, such as proliferation, apoptosis, and growth [68].

Using human prostate specimens and PCa cell lines, decreased DAB2IP is associated with PCa progression and induces G_0_/G_1_ cell cycle arrest while promoting cell apoptosis. Moreover, DAB2IP could suppress AKT and facilitate ASK1 activation under TNF-α treatment [19]. The PR domain within DAB2IP is exactly the binding site to p85. Noticeably, DAB2IP-mediated binding and inhibition of PI3K-AKT also contributes to ASK1 activation. Thus, DAB2IP is a scaffold protein capable of bridging both survival and death signal molecules, coordinating both PI3K-AKT and ASK1 pathways, which implies its role in maintaining cell homeostasis.

By scanning the DAB2IP protein sequence, Dai et al. identified two consensus Akt sites (RxRxxpS/T) located in the C terminus of DAB2IP at Serine-847 and Serine-907 [31]. Only Akt expression could increase phosphorylation of DAB2IP at S847, and the phosphorylated DAB2IP blocks interaction with H-Ras and TRAF2. Thus, Akt would inhibit DAB2IP, which would further fuel the Akt activity, and so on in a positive feedback loop.

#### 4.1.5 Androgen receptor signaling

Altered androgen receptor (AR), a typical transcription factor, is commonly associated with PCa progression. Data from clinical specimens and animal models clearly indicate an inverse correlation between AR and DAB2IP expression. DAB2IP could block AR nuclear translocation as well as AR transcriptional activities [69]. Moreover, the unique PR domain in DAB2IP is capable of competing with AR to form complex with c-Src in PCa cells, inhibiting c-Src and Erk signaling pathway. As a consequence the DAB2IP could block the genomic and non-genomic pathway of AR activation. DAB2IP can also suppress the activities of constitutively active AR splice variants.

### 4.2 Epithelial-to-Mesenchymal Transition (EMT)

The EMT is a highly conserved cellular program that allows polarized, immotile epithelial cells to convert to motile mesenchymal cells. This important development program is often activated during cancer invasion and metastasis [70].

#### 4.2.1 Glycogen Synthase Kinase (GSK)-3β-β-catenin signaling (Wnt pathway)

In canonical Wnt pathways, GSK-3β-mediated β-catenin degradation is inhibited, leading to accumulation of β-catenin in the nucleus. Nuclear β-catenin binds to members of the TCF/LEF family of transcription factors to promote EMT [71].

As mentioned above, DAB2IP functions as a tumor suppressor in cancer development with its cell-growth inhibition and pro-apoptosis. We first shifted emphasis to its role in cancer metastasis and found that the loss of DAB2IP expression initiates EMT in both human normal prostate epithelial and prostate carcinoma cells, as well as in clinical PCa specimens [34]. Conversely, restoring DAB2IP in metastatic PCa cells reversed EMT. In DAB2IP knockout mice, prostate epithelial cells exhibited elevated mesenchymal markers. Using a human prostate xenograft-mouse model, we observed that knocking down endogenous DAB2IP in human carcinoma cells led to the development of multiple lymph node and distant organ metastases. As for the mechanisms, we also found that DAB2IP modulates GSK-3β-β-catenin signaling through activation of GSK-3β by reducing Ser9 phosphorylation with the help of PP2A-DAB2IP complex. Moreover, the C2 domain was the key domain that interacts with both GSK-3β and PP2A, and facilitates GSK-3β activation to decrease nuclear β-catenin accumulation and its transcriptional activity.

#### 4.2.2 NF-κB signaling

NF-κB has a crucial role in EMT and also promotes tumor progression and metastasis in many cancers [72]. In primary human prostate immortalized epithelial cells (PrECs), DAB2IP ablation activated NF-κB and increased expression of NF-κB transcriptional targets. Conversely, DAB2IP reconstitution suppressed NF-κB and target genes in PC-3 cells [20]. Notably, the DAB2IP-S604A mutant, which is a point mutant in DAB2IP preventing TRAF2 binding, was defectively suppressing invasion and EMT. Since Ras fuels the NF-κB pathway [73], they further found that loss of DAB2IP induces the activation of Ras and NF-κB in PCa. Thus, DAB2IP functions as a signaling scaffold protein that coordinately regulates Ras and NF-κB through distinct domains to repress tumor growth and metastasis, respectively.

### 4.3 Angiogenesis

Angiogenesis, the process of new blood vessel formation, involved in many physiological and pathological settings such as ischemia, diabetes, atherosclerosis, and cancer [74]. Vascular endothelial growth factor, VEGF exerts its biologic effect through interaction with receptors present on the cell surface. Upon binding of VEGF to the extracellular domain of the receptor, dimerization and autophosphorylation of the intracellular receptor tyrosine kinases occur and a cascade of downstream proteins are activated [75].

In the research of *in vivo* functions of DAB2IP, Zhang et al. [76] created genetically deficient of the *DAB2IIP* gene mice (KO mice). KO mice exhibited dramatically enhanced angiogenesis in two models of inflammatory angiogenesis, one of which was associated with increased VEGF-VEGFR2 signaling. Consistent with this, VEGF-induced ear, cornea, and retina neovascularization were greatly augmented in KO mice. While over expression of DAB2IP, the enhanced retinal angiogenesis was markedly diminished. *In vitro*, VEGF-induced EC migration was inhibited by DAB2IP overexpression associated with decreased VEGFR2 signaling. It was further found that the VEGFR2-PI3K-DAB2IP forms a complex and plays a critical role at a late phase of VEGF response. DAB2IP combines with VEGFR2 and PI3K through different domains, C2 domain and PR domain respectively. Thus, *in vivo* and *in vitro* studies demonstrate that DAB2IP functions as an endogenous scaffold protein in inflammatory angiogenesis by suppressing VEGFR2-Akt-dependent signaling.

### 4.4 Radioresistance

When it comes to cancer management, radiation therapy has the advantage of being noninvasive and well tolerated. However, the rate of biochemical/clinical relapse for a significant number of patients, especially PCa, undergone radio-therapy unfortunately remains high [77]. One possible reason may be due to the intrinsic or acquired radioresistance of a subpopulation of tumors. Ionizing radiation (IR) causes several types of DNA damage, induces the formation of DNA-DNA and DNA-protein cross-links and causes single-(SSB) and double-stand breaks (DSB) [78]. Cell cycle checkpoint and apoptosis are both defense mechanisms to protect cells from DNA damage allowing them to repair genetic lesions [79, 80].

Kong and colleagues found that down-regulation of DAB2IP significantly enhances IR resistances in both PCa and normal prostate epithelial cells [81]. Knockdown of DAB2IP is associated with accelerated DSB repair kinetics, exhibits a robust early G_2_-M checkpoint and shows resistance to IR-induced apoptosis. Recently, we further found that Cytolethal distending toxin (CDT), a bacterial genotoxin secreted by *C. jejuni*, can induce cell cycle arrest at G2/M and apoptosis in DAB2IP-deficinted PCa cells exhibiting a radio-resistant phenotype [82]. The mechanisms are attributed to the degradation of host cell DNA and cell cycle arrest, resulting in an increased induction of apoptosis.

DNA-PKcs, the catalytic subunit of DNA-dependent protein kinase, plays a dominant role in nonhomologous end joining (NHEJ)-mediated DSB repair, genomic intergerity, and maintaining telomere stability [83, 84] and is upregulated in various cancers [85]. Inhibitors of DNA-PKcs, such as NU7441 have been developed to enhance radiation treatment-based local tumor control [86]. With the goal of developing strategies to overcome radioresistance of DAB2IP-negative PCa and improve the efficacy of RT in PCa, Yu et al. clearly found that adjuvant treatment with NU7441 can overcome PCa radioresistance which is caused by loss of DAB2IP [36].

As radiation therapy continues expand its role in bladder cancer (BCa) treatment, DAB2IP shows the similar potent tumor suppressor gene property [87, 88]. Tingting et al. found that decreasing DAB2IP expression induces radio-resistance in BCa and associates with increased ataxia-telangiectasia mutated gene (ATM) expression [88]. Phosphorylation or inhibitor (KU55933) of ATM could be utilized to enhance DAB2IP knockdown cells sensitivity to irradiation.

### 4.5 Autophagy

According to former study, loss of DAB2IP resulting in radioresistance *in vitro* [81, 89], they subsequently performed a retrospective cohort study and determined that loss of DAB2IP leads to significantly increased rate of biochemical failure after radiotherapy [36].

Autophagy is a lysosomal degradation pathway that eliminates damage or potentially dangerous proteins and organelles under adverse conditions to protect organisms from metabolic stress [90]. Many studies have shown that cancer cells use autophagy as an adaptive and context-dependent system to overcome radiotherapeutic stress [91]. In response to radiation, DNA-PKcs involved in autophagy and inhibition of DNA-PKcs sensitizes cells to IR-induced autophagic cell death [92]. This means that NU7441 treatment can promote both IR-induced and basal level of cell autophagy, while overexpression of DAB2IP attenuated IR and NU7441 induced autophagy [36]. There is controversial opinion of the function of Akt-mTOR-S6 pathway regulating autophagy [90, 93]. To explore the possible mechanism underling the regulation of DAB2IP in autophagy, they also found that mTOR-S6K pathway was inactivated in DAB2IP-expressing PCa cells. Thus, it is conceivable that DAB2IP inhibits autophagy through suppressing mTOR-S6K pathway.

### 4.6 Chemo-resistance

Understanding the mechanisms for chemo-resistance of cancer is pivotal, because cancer cells eventually develop chemo-resistance [94].

When we discuss the radio-resistance of DAB2IP-deficient PCa cells, these cells show less sensitive to Epothilone B (EpoB), which is a commonly used chemotherapeutic drug clinically [89]. Further studies tried to test the chemo-sensitivity among other chemotherapeutics (i.e. Docetaxel, Gemcitabine, Istodax and EPoB) in PCa cells [95]. As a result, DAB2IP-KD cells showed significantly higher resistance to all four drugs, while stable DAB2IP-expressing subline becomes more sensitive to chemotherapeutic agents.

Secretory clusterin (sCLU) is a stress-activated cytoprotective chaperone up-regulated by many varied anti-cancer therapies to confer treatment resistance [96]. CLU protein and mRNA expression as well as the promoter activities inversely correlated with DAB2IP expression levels [95]. Since sCLU was regulated by IGF-1R/Src/Erk/Egr-1 signaling where the early growth response-1 (Egr-1) is a key transcription factor controlling CLU gene promoter activity [97], they subsequently showed that DAB2IP shows inhibitory effect on Egr-1expression via crosstalk between Wnt-β-catenin and IGF-1/IGFR signaling.

### 4.7 Cancer stem cells

Cancer stem cells (CSCs), which make up a small fraction of cancer cells, are responsible for the initiation, progression, local and distant recurrence/metastasis of cancer, also for the failure of chemo- and radiotherapy. CSCs exhibit many similar properties as normal stem cells do, such as pluripotency, self-renewal and slow proliferation [98, 99].

Yun and colleagues demonstrated that loss of DAB2IP enriches CSCs characteristics in human PCa cells [35]. Even injecting very low cell number of KD cells such as one single cell into immune-deficient mice could develop subcutaneous tumors. By screening the various stem cells markers, they discovered that DAB2IP suppress CD117 in both protein and transcriptional level. DAB2IP could facilitate the binding of GATA-1 to CD117 gene promoter while repressing CD117 transcriptional activity. LZ domain within the C-terminal end of DAB2IP is the key domain of interacting with GATA-1. Since CD117 is a receptor tyrosine kinase, it may regulate many downstream effectors including PI3K, MAPK, PKC and JAK-STAT pathways [100]. DAB2IP could directly inhibit PI3K-Akt-mTOR signaling pathway that increases c-myc protein to activate ZEB1 gene expression leading to elevated CSC phenotypes. Thus, DAB2IP appears to have a homeostatic role in modulating stemness of PCa exhibiting some basal cell phenotypes via CD117-ZEB1 signal axis.

## 5 CONCLUSIONS AND PERSPECTIVES

Almost thirteen years ago, DAB2IP was firstly identified as a DOC-2/DAB2 interactive protein with the growth-inhibitory effect in prostate cancer by our group. Since then, the functions of DAB2IP have extended to regulating cell proliferation, survival, apoptosis, epithelial-to-mesenchymal transition (EMT), cancer stem cell phenotype, radiation and chemotherapy resistance by our group and other groups. However, other functions of DAB2IP in tumor progression are still uncertain. For instance, DAB2IP inhibits VEGFR2-mediated adaptive angiogenesis in vascular ECs [76], while its role in tumor-associated angiogenesis is not well characterized. Metabolic reprogramming is considered a hallmark of cancer [99], and appears to be an area of accelerated research over the last decade [101]. Many target gene and signal pathways DAB2IP involved have been identified and it is interesting to explore whether DAB2IP contribute to reprogrammed cancer cell metabolism and the possible mechanism should be elucidated in the future.

More importantly, the mechanism of how DAB2IP is regulated is still one of the most important questions to be answered. As shown before, DAB2IP is frequently silenced by DNA methylation, histone methylation and acetylation in aggressive human tumors [10, 21–23, 31, 88]. Nonetheless, the promoter of DAB2IP may not be methylated and/or acetylation in some tumors, and alternative mechanisms might exist to interfere with its functions. Recently, Di Minin and colleagues showed that DAB2IP can be functionally inactivated by physical interaction with mutant p53 proteins, with implications for the response of cancer cells to inflammatory cytokines [62]. It is reasonable to speculate that whether DAB2IP can be functionally inactivated with wild-type p53 and the possible mechanisms and functions underlying this interaction. Furthermore, our group demonstrated that DAB2IP expression can be regulated by Skp2-mediated proteasome degradation [9], so others proteasome degradation mechanisms are still remain to be resolved. MicroRNAs (miRNAs) are 20–25-nt-long highly conserved non-coding RNAs that bind to sequences within the 3′ untranslated region (3′ UTR) of mRNAs and post-transcriptionally regulate the expression of target genes [102]. Recently, Xu Y et al. found that DAB2IP is a direct target of miR-889 in esophageal squamous cancer [103], while others MicroRNAs involved in regulating DAB2IP expression will be discovered in the future. Moreover, Long non-coding RNAs (lncRNAs) defined as transcripts longer than 200 nucleotides (nt) non-coding RNA, function in a wide range of biological processes and can regulate gene expression in cis or in trans by diverse mechanisms [104]. The possible regulation between lncRNAs and DAB2IP, however, has not been reported. Hence, in the next few years more and more research will shed light on the function and regulation of DAB2IP and provide insights into therapies which could be used to target these cancers.
